# A case report of a cat infected with European bat lyssavirus type 1, the Netherlands, October 2024

**DOI:** 10.2807/1560-7917.ES.2025.30.10.2500154

**Published:** 2025-03-13

**Authors:** Phaedra Eblé, Aldo Dekker, Sanne van den End, Vanessa Visser, Marc Engelsma, Frank Harders, Lucien van Keulen, Erik van Weezep, Melle Holwerda

**Affiliations:** 1 Wageningen Bioveterinary Research (WBVR), Lelystad, the Netherlands; 2 The Netherlands Food and Consumer Product Safety Authority (NVWA), Utrecht, the Netherlands

**Keywords:** Rabies, European bat Lyssavirus, zoonosis, cat, bat, EBLV-1

## Abstract

In October 2024, an infection of European bat lyssavirus type 1 was confirmed in a domestic cat in the Netherlands. Several weeks before, the owners had found a dead bat considered to be caught by the cat. Nine persons exposed to the cat received post-exposure prophylaxis and four domestic animals from the same household were quarantined. This report stresses the need for vigilance for rabies in domestic animals in countries where lyssavirus infections in bats are endemic.

European bat lyssaviruses (EBLV) are present in some bat species in the Netherlands. Mammals, including humans, can serve as natural hosts for lyssaviruses. Without proper prophylaxis and/or treatment, an infection can be fatal. Here, we present detection of EBLV type 1 (EBLV-1) in a cat showing altered behaviour after an assumed contact with a bat.

## Case report

On 25 October 2024, laboratory tests confirmed EBLV-1 (lyssavirus Hamburg) infection in a domestic cat with outdoor access in the Netherlands. According to the owner, the cat started to show abnormal behaviour on 22 October 2024. One day later, the cat became aggressive and was taken to the local veterinarian who prescribed sedatives and painkillers. Because of the aggressive behaviour, the cat was isolated. The owners of the cat told the veterinarian that a dead bat had been found in the house approximately 3.5 weeks earlier, presumably caught by the cat. Due to the lack of improvement in the behaviour of the cat with the medication and the characteristic clinical symptoms along with a history of contact with a bat, a suspicion of a possible rabies infection was raised by the veterinarian. The case was reported to the Dutch competent authorities, the Netherlands Food and Consumer Product Safety Authority (NVWA), the Public Health Service (GGD) and the National Institute for Public Health and the Environment (RIVM) on 24 October. The bat had been disposed of and was not sent in for lyssavirus diagnostics nor had the bat species been identified. The cat had not been vaccinated against rabies.

## Laboratory analyses

**Figure 1:**
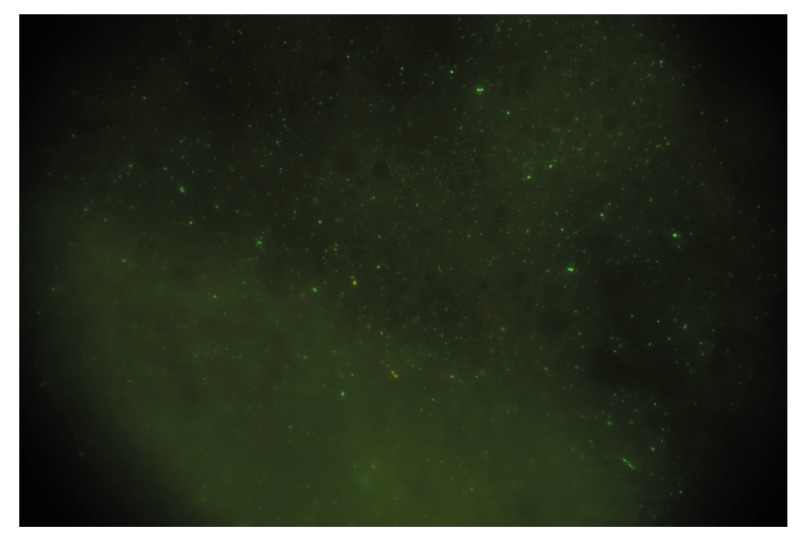
Direct fluorescent antibody test of brain tissue from a cat infected with European bat lyssavirus 1, Netherlands, October 2024

On 25 October, the cat was euthanised and sent to the National Reference Laboratory (NRL) for animal rabies of the Netherlands, Wageningen Bioveterinary Research (WBVR). Brain tissue of the cat tested positive in the direct fluorescent antibody test (DFA) [1], (Figure 1). Also, two genotype-specific real-time PCR (RT-PCR) tests were conducted on the brain material. The RT-PCR test for classical rabies virus (RABV) [2] tested negative, whereas the RT-PCR test for EBLV-1 (in-house PCR test) tested positive with a quantification cycle (Cq) value of 14.

Direct fluorescent antibody test of the cat showing rabies-specific apple-green fluorescent spots.

**Figure 2:**
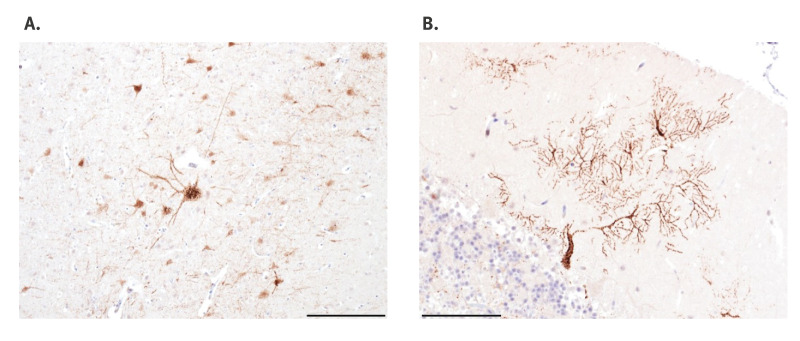
Positive immunohistochemical staining for the rabies nucleocapsid protein in neurons of the cerebral cortex (A) and in the Purkinje cells and their dendrites in the cerebellar cortex (B) of a cat infected with European bat lyssavirus, Netherlands, October 2024

Follow-up diagnostics at WBVR was performed. A sample from the salivary gland and a swab sample from the mouth of the cat tested positive for EBLV-1 in the RT-PCR (Cq values of 24 and 26, respectively). Histopathology of formalin-fixed and paraffin-embedded sections of the brain showed a viral encephalitis with positive immunohistochemical staining against the rabies nucleocapsid protein (Figure 2).

A. Bar = 200 μm.

B. Bar = 100 μm.

Dark brown staining indicates the presence of rabies virus antigen.

**Figure 3:**
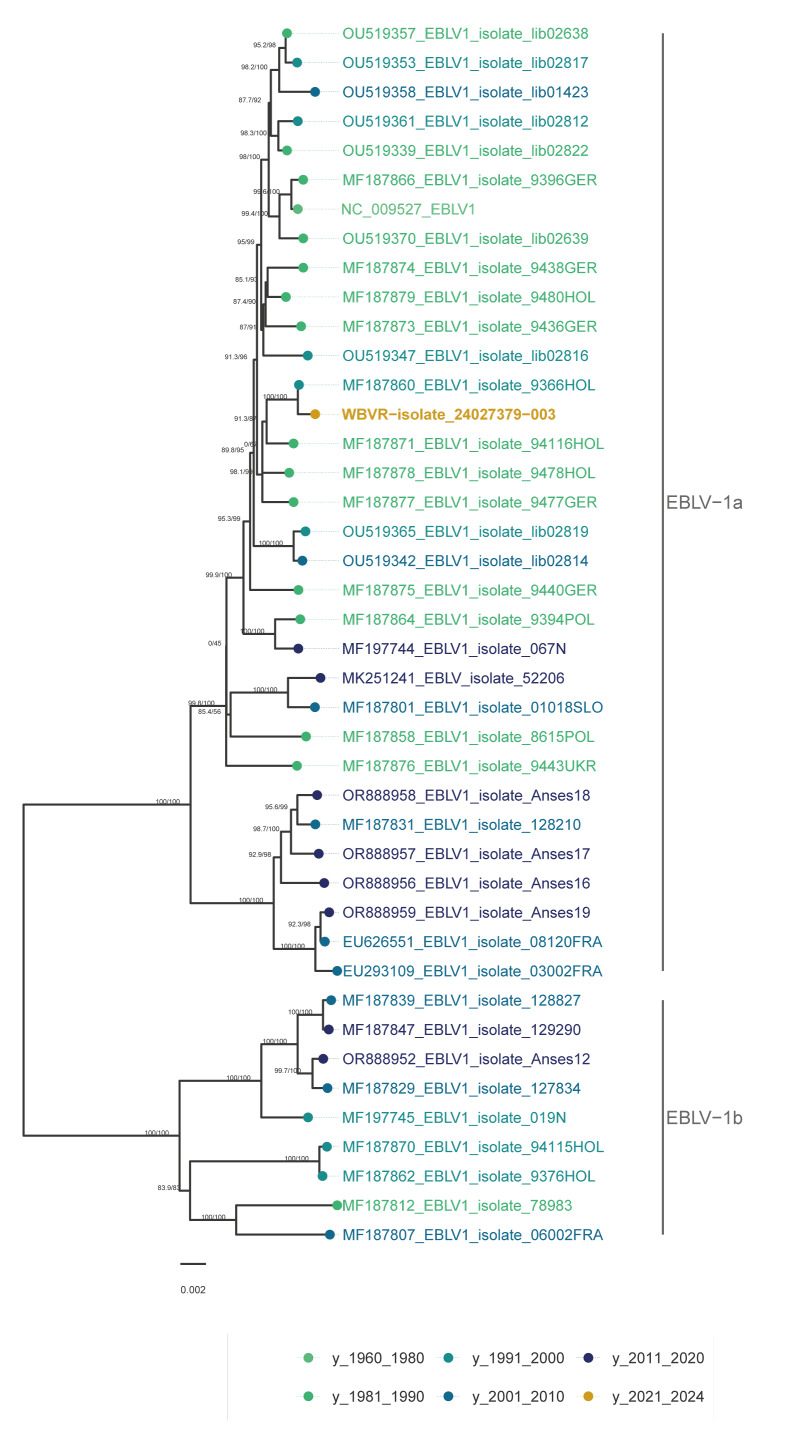
Maximum likelihood phylogenetic tree reconstructed of sequences from the public NCBI database of European bat lyssavirus 1, 1960–2024 (n = 41)^a^

EBLV: European bat lyssavirus; NCBI: The National Center for Biotechnology Information; y: year.

^a^ The sequence highlighted in yellow represents the virus isolate sequenced from this study.

The entire genomic sequence was determined using Oxford Nanopore Technologies (Oxford, the United Kingdom) [3]. Phylogenetic analysis was done by comparing with 41 EBLV-1 sequences available in the National Center for Biotechnology Information (NCBI; https://www.ncbi.nlm.nih.gov/) database and showed that the cat was infected with an EBLV-1a strain that had 99.86% homology to a virus detected in 1987 in a bat in the Netherlands (Figure 3) [4].

## Public health measures

Human and animal contacts were traced by the GGD and NVWA. Nine persons were vaccinated against classical rabies (WHO Essen scheme) [5]. Seven of these persons were bitten or scratched by the cat or had injuries that possibly had contact with saliva of the cat, and they also received human rabies immunoglobulin (HRIG) [5]. Additionally, two dogs residing in the same house as the cat and two horses in a stable at the same address, were quarantined for 6 months. The animals were taken care of by rabies-vaccinated people and were not allowed to have contact with other pets, hobby animals or livestock. After consultation with the town mayor and the local veterinarians, the GGD and NVWA informed nearby residents of the (rural) municipality by a letter delivered to their homes. The letter contained information about the case, rabies virus and rabies infection, also in bats, including links to relevant websites. Also, a request was made for special attention to behavioural changes in pets and to report these immediately to a veterinarian. Pet owners were informed via the two local veterinarians.

## Discussion

The Netherlands has been free from RABV for decades. However, EBLV is endemic in two of the seventeen bat species present in the Netherlands: EBLV-1 in serotine bats (*Eptesicus serotinus*) and EBLV-2 in pond bats (*Myotis dasycneme*) [6]. The last time EBLV-2 was detected was in 1993, but EBLV-1 is detected annually in circa 1–2 of 50 bats sent to the NRL from various places of the Netherlands. Between 1984 and 2003, 251 (20.6%) of 1,219 serotine bats in the Netherlands tested positive for lyssavirus antigen [6]. However, the analysed bats in that study were predominantly sick or dead animals, and therefore the actual prevalence of EBLV-1 in the healthy Dutch bat population is not known but most likely lower.

Like other lyssaviruses, EBLV is probably able to infect all mammals, including humans, and an infection can be fatal. In the Netherlands, bats that have had direct contact (bite, scratch, lick) with a human, and are available for testing, are tested within 24 h for the presence of lyssavirus, to be able to treat the exposed person timely with post-exposure prophylaxis (PEP). In contrast, laboratory tests are not performed immediately (within 24 h) of bats sent to the NRL after contact with pets, instead, vaccination against rabies is recommended for cats and dogs that have had direct contact with bats.

Despite the presence of EBLV in European bats, reports of EBLV in non-flying mammals are scarce. Transmission of EBLV to mammals has been described in Europe: to a stone marten in Germany, sheep in Denmark and cats in France [7]. In addition to the findings in cats in 2003 and 2007 [7], EBLV was detected in a third cat in France in 2020 (personal communication: Emmanuelle Robardet, the European Union Reference Laboratory (EURL) for animal rabies, ANSES, France, October 2024). Fatal cases of EBLV infections in humans have been described in 1985 in Russia and in Finland, in 2002 in Scotland and in Ukraine and in 2019 in France, all after (supposed) contact with bats [8]. Transmission from other mammals than bats to humans has not been described. Previously, cats were considered dead-end-hosts for EBLV infections since only a small amount of EBLV virus particles could be detected in the samples [7]. However, in our case report, the infected cat showed clinical signs of rabies, and a high viral load was detected in the samples from brain, salivary gland and mouth swab of the cat. Therefore, we consider that this cat may have been infectious and probably capable of transmitting the virus to other hosts.

The sequence of the virus from the cat had a high homology to an EBLV-1a detected in a bat in 1987, found within a 50 km distance from the cat, which indicates a high genomic stability of the virus. Additionally, it is unlikely that this strain was a new variant or that the virus had adapted in either the bat population or in or towards its new host.

In 2020, a spillover of a West Caucasian bat virus (WCBV) to a cat in Italy was reported [9]. In contrast to WCBV, EBLV is within the same phylogroup of lyssaviruses (I) as RABV. Therefore, in the current investigation, vaccination against RABV could be implemented for exposed persons, knowing that this would be effective as protective measure. The spillover of EBLV-1 from a bat to a domestic cat and the following exposure of people highlights the rare but possible hazard of exposure of humans to a lyssavirus via their pet.

## Conclusion

Confirmation of an EBLV-1 infection in a domestic cat highlights that vigilance for rabies is necessary, also in countries free from classical rabies but where EBLV is endemic in the bat population. To avoid further transmission of the virus to other (domestic) animals or humans, recognition of the clinical disease (particularly in predators such as cats), rapid laboratory diagnosis and timely implementation of control measures are of paramount importance.
